# Fully automated spleen segmentation predicts progression-free survival in HCC patients following transarterial radioembolization

**DOI:** 10.1007/s00259-026-07792-8

**Published:** 2026-02-17

**Authors:** Lisa Steinhelfer, Friederike Jungmann, Lukas Endroes, Manuel Nickel, Noemi Schweizer, Ursula Ehmer, Bernhard Haller, Robert Walter, Christopher Spaeth, Henrik Einwächter, Jannis Bodden, Karina Knorr, Matthias Eiber, Rickmer Braren

**Affiliations:** 1 Department of Diagnostic and Interventional Neuroradiology, Technical University of Munich, School of Medicine and Health, TUM University Hospital, Klinikum Rechts der Isar Ismaninger Str. 22, Munich, 81675 Germany; 2 Department of Diagnostic and Interventional Radiology and Nuclear Medicine, University Medical Center Hamburg-Eppendorf, Hamburg, Germany; 3 Department of Nuclear Medicine, Technical University of Munich, School of Medicine and Health, TUM University Hospital Munich, Munich, Germany; 4 Department of Radiology, Technical University of Munich, School of Medicine and Health, TUM University Hospital, Munich, Germany; 5 Chair for AI in Healthcare and Medicine, Technical University of Munich (TUM) and TUM University Hospital, Munich, Germany; 6 TUM School of Medicine and Health – Clinical Department of Internal Medicine II, TUM University Hospital, Munich, Germany; 7 Bavarian Cancer Research Center (BZKF), Erlangen, Germany; 8 German Cancer Consortium (DKTK), partner site Munich, a partnership between DKFZ and School of Medicine, Technical University of Munich, Munich, Germany

**Keywords:** HCC, TARE, AI-based volumetry, Spleen volume, Imaging biomarker

## Abstract

**Purpose:**

Transarterial radioembolization (TARE) is a well-established treatment for unresectable hepatocellular carcinoma (HCC), though its effects on non-tumorous tissue remain a concern. In particular, the prognostic relevance of splenic volume changes after TARE is not fully understood. This study aimed to assess imaging-derived markers—specifically splenic volume dynamics—as predictors of disease progression.

**Methods:**

We retrospectively analyzed laboratory and imaging data from 73 patients with histologically or imaging-confirmed HCC who underwent TARE with Yttrium-90 (^90^Y) at our institution between January 2012 and September 2022. Inclusion criteria were age ≥ 18 years, availability of baseline and 3-month follow-up imaging, and complete clinical documentation. Patients undergoing liver resection, transplantation, or additional therapies during follow-up were excluded.

**Results:**

A relative increase in splenic volume at 3 months was the only independent predictor of progression-free survival (PFS), yielding a ROC-AUC of 0.86 (95%-CI: 0.76–0.95). An increase of 18% or more most accurately identified patients with early disease progression (< 12 months) with a sensitivity 0.74 and specificity 0.97, outperforming conventional clinical and laboratory parameters, including two-dimensional craniocaudal spleen measurements.

**Conclusions:**

Automated splenic volumetry showed superior prognostic value over traditional markers in HCC patients treated with TARE. A post-treatment increase in spleen volume represents an additional, robust, and readily accessible imaging biomarker for early risk stratification and individualized treatment planning.

**Supplementary Information:**

The online version contains supplementary material available at 10.1007/s00259-026-07792-8.

## Introduction

The prevalence and mortality of hepatocellular carcinoma (HCC) are increasing globally, making it the fourth leading cause of cancer-related deaths and accounting for 85–90% of all primary liver cancers [1]. Unlike many other cancers, the risk factors for HCC are well established. HCC commonly occurs in patients with advanced hepatic fibrosis or cirrhosis, primarily resulting from chronic liver disease. Hepatitis B (HBV) and hepatitis C (HCV) infections are two of the leading causes of liver damage resulting in HCC [2–4]. In addition, metabolic dysfuction-associated steatotic liver disease and alcohol-associated liver diseases are the fastest rising causes of HCC-related death [5]. Although surgery offers the best chance for cure, HCC carries a poor prognosis with the majority of cases diagnosed at an intermediate or advanced stage where treatment is considered non-curative [6].

Transarterial radioembolization (TARE) with yttrium-90 (⁹⁰Y) microspheres is a form of intra-arterial brachytherapy that delivers high-dose radiation directly to liver tumors via the hepatic artery. Retrospective series and large cohort studies have demonstrated a favorable safety profile and encouraging outcomes in terms of local disease control and overall survival in patients with unresectable HCC, including those with portal vein thrombosis and intermediate- to advanced-stage disease [7–11].

Complications primarily arise from excessive irradiation of non-target liver tissue. In cirrhotic livers, the distribution of microspheres can be significantly altered due to vascular changes associated with cirrhosis, such as arterio-portal and arterio-venous shunts. These alterations affect the radiation dose absorbed by both tumor and non-tumor liver tissue, influencing treatment tolerance and effectiveness. Additionally, the reduced functional reserve in cirrhotic livers increases the risk of liver failure, particularly after major surgery or in response to stressors such as viral or toxic hepatitis and external irradiation [12]. Combined with radiation-induced damage to liver cells and blood vessels, these factors make cirrhotic patients more vulnerable to clinically significant liver toxicity following radioembolization compared to those with non-cirrhotic livers [13].

Damage to non-tumorous liver parenchyma can result in hepatic volume changes, liver fibrosis, and increased splenic volume [14, 15]. These changes may result from parenchymal contraction driven by oxidative stress and inflammatory cytokine activation, with subsequent coagulation cascade involvement, leading to portal hypertension and splenic congestion - processes that may be further aggravated in patients with pre-existing cirrhosis [16].

Previous studies have demonstrated that increased splenic volume is closely associated with severe liver damage. It has emerged as a highly sensitive prognostic marker for patients with HCC undergoing resection or tumor ablation, effectively predicting the severity of liver fibrosis and portal hypertension across various stages of liver disease [17–21].

However, manual assessments of splenic volume on cross-sectional CT images is labor-intensive and prone to interrater variability [22]. Consequently, such assessments are impractical for routine clinical use. Fortunately, recent advancements in artificial intelligence, particularly in deep learning, have facilitated automated organ segmentation and volume assessments. These algorithms seamlessly integrate into clinical workflows in real-time [23]. Therefore, splenic volume may serve as a readily accessible prognostic factor for treatment planning and post-TARE follow-ups. This study aims to validate the role of hepatic and splenic volume as imaging biomarkers for survival prediction and to explore the potential as indicators for hepatic decompensation in patients with HCC undergoing TARE.

## Materials and methods

### Patients

All patients underwent pretreatment evaluation, including clinical history, physical examination, laboratory tests, and baseline imaging. Inclusion criteria were age ≥ 18 years; histologically or imaging-confirmed HCC per EASL guidelines [24–26]; Eastern Cooperative Oncology Group (ECOG) performance status 0–2; and total bilirubin < 3.0 mg/dL. Portal vein thrombosis (PVT) and limited extrahepatic disease were no exclusion criteria. Eligible patients had baseline and follow-up CT and/or MRI; available lab data; and a minimum follow-up of 3 months. Patients undergoing liver resection, transplantation, or additional therapies during follow-up were excluded.

Treatment decisions were made by consensus in a multidisciplinary tumor board including hepatologists, medical oncologists, transplant surgeons, and interventional radiologists. All patients underwent preprocedural angiography and simulation with technetium-99 m-labeled macroaggregated albumin (⁹⁹ᵐTc-MAA) to assess hepatic arterial anatomy and evaluate hepatopulmonary and gastrointestinal shunting. Prophylactic embolization was performed when indicated to prevent non-target radiation (gastroduodenal artery, *n* = 1; right gastric artery, *n* = 3; other extrahepatic vessels, *n* = 4). All patients were treated with ^90^Y resin microspheres (SIRTeX Medical, Sydney, Australia) using body surface area to calculate liver mass according to the technique previously described in detail [27, 28]. TARE was performed either as first-line therapy (*n* = 42) or after prior treatments, including resection (*n* = 11), TACE (*n* = 8), RFA (*n* = 4), checkpoint inhibitors (*n* = 5), or chemotherapy (*n* = 3). Ethical approval for this retrospective analysis was obtained from the local institutional review board (reference 87/18S). Patient selection is illustrated in Supplementary Figure S1.

### Image segmentation

**Fig. 1:**
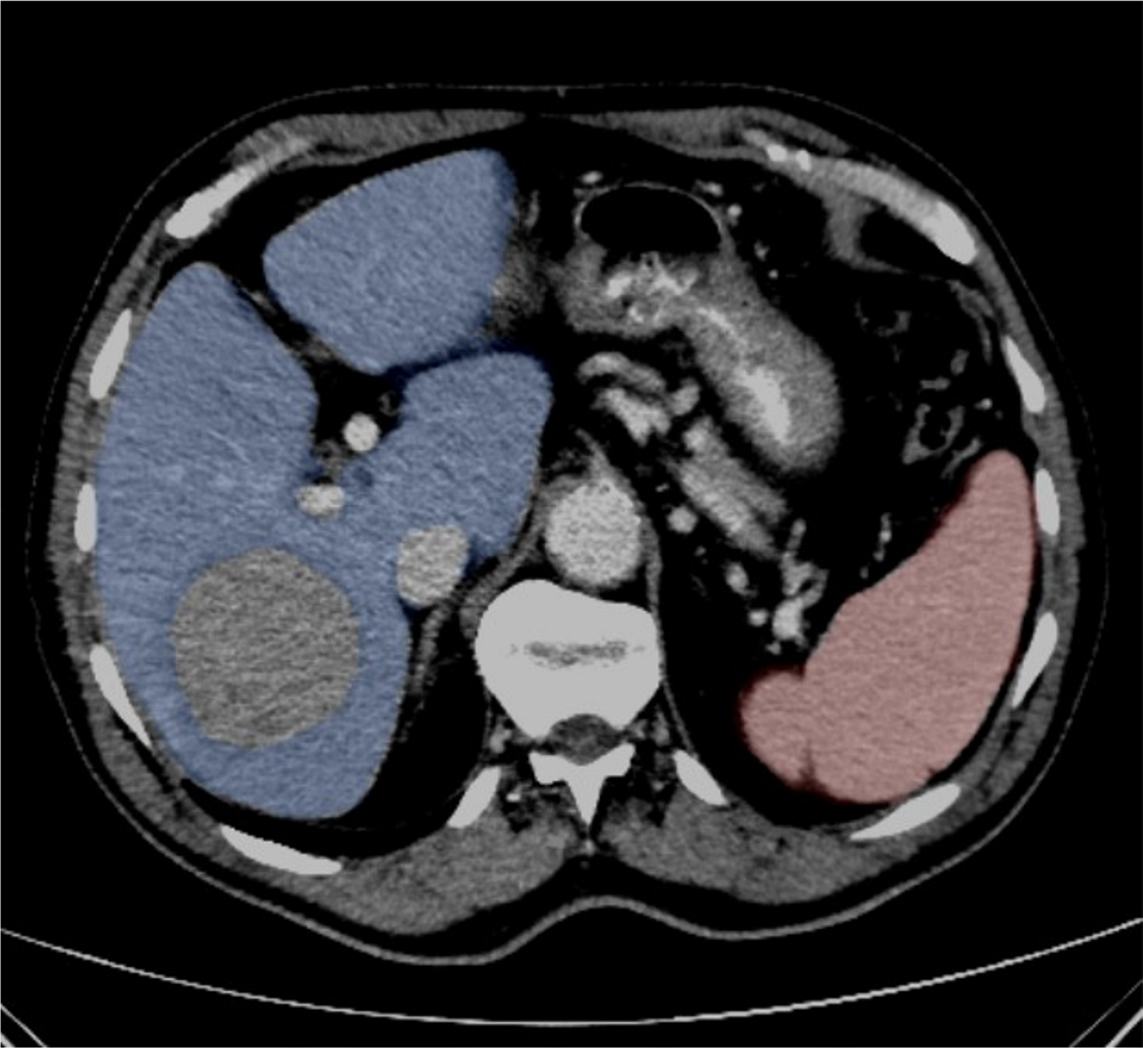
Axial CT image illustrating segmentation of functioning liver tissue (blue area) and, spleen volume (red area), excluding cysts, tumor lesions and major vessels, using TotalSegmentator

Diagnostic CT scans were used to assess hepatic and splenic volume before treatment and at 3-months follow-up. In order to automatically obtain the organ volume from CT scans, a publicly available deep-learning segmentation model (TotalSegmentator) was employed [29]. Organ volume was determined as the healthy liver and spleen parenchyma, excluding cysts, tumor lesions and major vessels (Fig. 1). Healthy hepatic parenchyma volume was calculated as the difference between total liver volume and tumor volume. The algorithm was applied to contrast-enhanced and/or native axial CT images with a slice thickness of ≤ 5 mm. For functional assessment, regions of interest (ROIs) were manually delineated for the entire liver and the target tumor on every axial slice of ^99m^Tc-MAA SPECT/CT, using angiography and contrast-enhanced CT for anatomical guidance. Necrotic tumor components were excluded from the analysis. Total counts derived from the SPECT/CT ROIs were used to estimate the tumor-to-normal liver ratio (TNr), calculated according to the formula described in the European Association of Nuclear Medicine guidelines [30]. Each segmentation was manually reviewed by two board-certified radiologists: L.S., with 5 years of experience, and R.B., an attending radiologist with 14 years of experience, and adjustments were made to the automatically measured contour if necessary.

### Follow-up

Biochemical tests, including alanine aminotransferase (ALT), aspartate aminotransferase (AST), γ-glutamyl transferase (GGT), albumin, bilirubin, hemoglobin (Hb), white blood cell (WBC), and platelet (PLT) counts, were regularly monitored. Additionally, the Child-Pugh score, MELD score, and ALBI score were assessed at baseline and at 3 months post-TARE, while BCLC stage was determined at baseline. Venous blood samples were collected following standard hospital protocols. Changes in these biochemical markers were evaluated at 3 months after treatment. The values were compared to baseline, and their relative changes were correlated with hepatic and splenic volumes. The primary endpoint was progression free survival (PFS), defined as the time from TARE to progressive disease.

### Statistical analysis

Statistical analyses were performed using Python 3.11.0 and R 4.5.0. All tests were performed two-sided using a level of significance of α = 0.05. Data are presented descriptively as mean values with standard deviation (SD) or as median and interquartile range (IQR). Progression free survival (PFS) was calculated starting at the time patients received TARE treatment and using follow-up and progression dates from our hospital database and interdisciplinary tumor board. Due to the high number of clinical and imaging parameters collected for each patient, univariate pre-selection was performed on all features using univariate Cox’s Proportional Hazards model with a cut-off of *p* < 0.1. Prior to inclusion in the multivariate model, the selected features were assessed for multicollinearity using variance inflation factors (VIFs). Features with a VIF greater than 5 were considered collinear and excluded from further analysis. The final multivariate Cox model was then fit using the remaining features, after confirming that the proportional hazards assumption was satisfied for each [31]. 95% confidence intervals were calculated for all hazard ratios. Receiver operating characteristic analysis and Youden index were used to determine a specific cutoff value of changes in spleen volume to distinguish between patients who had an PFS of more than a year and patients who did not.

## Results

### Patient characteristics

The baseline characteristics of patients undergoing TARE are summarized in Table 1. We identified 73 patients with confirmed HCC (of whom 10 patients (13.7%) had an encapsulated nodular type, 38 (52.1%) a simple nodular type with extranodular growth, 10 (13.7%) a confluent multinodular type, and 15 (20.5%) an infiltrative type) who underwent TARE with Yttrium-90 (⁹⁰Y) resin microspheres (SIR-Spheres; Sirtex Medical, Sydney, Australia) between January 2012 and September 2022. Of the 73 patients included, 57 were male (78.1%) and 16 were female (21.9%), with a median age of 72 years (IQR 59–77). The primary causes of liver disease included chronic hepatitis C infection (19.2%), alcohol-related liver disease (47.9%), chronic hepatitis B infection (11%), and metabolic dysfunction–associated steatotic liver disease (10%). All patients received a single TARE treatment, with a median pre-treatment lung shunt of 7% (IQR 5% – 10%) and a median administered TARE dose of 1.80 GBq (IQR 1.45–2.47). Treatment was administered to the left hepatic lobe in 34 patients (46.6%) and to the right lobe in 39 patients (53.4%). Embolization strategies included lobar TARE in 48 patients (65.7%), segmental TARE in 21 patients (28.8%), and subsegmental TARE in 4 patients (5.5%). Liver function was assessed using the Child–Pugh classification, with 58 patients (79.5%) in class A and 15 patients (20.5%) in class B. Based on the albumin–bilirubin (ALBI) score, 49 patients (67.1%) were classified as grade 1, 23 (31.5%) as grade 2, and 1 (1.4%) as grade 3. At baseline, BCLC staging indicated that 1 patient (1.4%) was classified as stage A, 63 patients (86.3%) as stage B, and 9 patients (12.3%) as stage C. Overall, the study cohort predominantly consisted of patients with intermediate-stage HCC and preserved liver function. A total of 146 CT scans were analyzed, including 73 baseline and 73 follow-up scans. The TotalSegmentator AI algorithm successfully segmented the liver and spleen in 141 of 146 scans (96.6%). Manual correction was required in only 5 cases (3.4%) - three due to under-segmentation and two due to over-segmentation, such as misidentification of adjacent structures like the portal vein. The median (IQR) baseline spleen volume was 369.3 (230.8–536.4) ml, and the median (IQR) craniocaudal diameter was 111.0 mm (99–134). The median (IQR) baseline hepatic volume was 1625.3 (SD 548.2) ml. The median percentage increase in spleen volume (%-SV) from baseline was 4.9% (IQR 0.7% − 34.7%) at 3 months, while the corresponding median percentage decrease in liver volume (%-LV) was − 1.7% (IQR − 4.9% − 1.4%).

**Table 1:** Baseline characteristics of patients included in the study (*n* = 73). +Values are presented as median with the interquartile range presented in parenthesis. *For categorical features, absolute value counts are given with percentage in parenthesis. Abbreviations: ALD = Alcoholic liver disease, MASLD = Metabolic dysfunction-associated steatotic liver disease, PLT = Platelet Count, AST = Aspartate Aminotransferase, ALT = Alanine Tansaminase, GGT = Gamma-Glutamyl Transferase, AFP = Alpha-fetoprotein

Clinical Characteristic	Value
Median Age^+^ (years)	72 (59–77)
Sex	
Male^*^	57 (78.1)
Female^*^	16 (21.9)
Etiology of Liver Cirrhosis	
ALD^*^	35 (47.9)
Hepatitis C^*^	14 (19.2)
Hepatitis B^*^	8 (11)
MASLD^*^	7 (9.6)
Unknown/Other^*^	9 (12.3)
Baseline Child-Pugh Score	
A^*^	58 (79.5)
B^*^	15 (20.5)
C^*^	0 (0)
Baseline ALBI Grade	
Grade 1^*^	49(67.1)
Grade 2^*^	23 (31.5)
Grade 3^*^	1 (1.4)
Baseline MELD Score^+^	8 (7–10)
Baseline BCLC Stages	
0	0 (0)
A^*^	1 (1.4)
B^*^	63 (86.3)
C^*^	9 (12.3)
D^*^	0 (0)
Baseline Tumor Volume^+^ [ml]	470 (314–625)
Tumor Growth Pattern	
Unifocal^*^	30 (41.1)
Multifocal^*^	36 (49.3)
Diffuse^*^	7 (9.6)
Morphology	
Encapsulated nodular^*^	10 (13.7)
Simple nodular type with extranodular growth^*^	38 (52.1)
Confluent multinodular^*^	10 (13.7)
Infiltrative	15 (20.5)
Baseline Albumin Level^+^ [g/l]	4.1 (3.8–4.4)
Baseline Bilirubin Level^+^ [mg/dl]	0.7 (0.5–0.9)
Baseline PLT^+^ [1000/l]	179 (124–243)
Baseline AST Level^+^ [U/l]	54 (40–74)
Baseline ALT Level^+^ [U/l]	40 (28–55)
Baseline GGT Level^+^ [U/l]	193 (102–328)
Baseline AFP^+^ [ng/ml]	25.7 (6.8–324.9.8.9)
Progression Free Survival^+^ [months]	12.6 (5.8–20.0)
TARE Activity [GBq]^+^	1.8 (1.5–2.5)
Embolization strategies	
Lobar^*^	48 (65.7)
Segmental^*^	21 (28.8)
Subsegmental^*^	4 (5.5)

### Survival analysis

All patients included in our study experienced an oncologic progression during the follow-up interval with a median PFS of 12.6 (IQR 5.8–20.0) months. 39 patients (53.4%) had a progression-free survival of ≥ 12 months, whereas 34 patients (46.6%) experienced progression within 12 months.

Univariate Cox proportional hazards analysis revealed 8 of 59 features tested to be significantly associated with PFS (*p* < 0.1), which are shown in Table 2. In detail, these were baseline ALT and MELD score, the change of MELD and Child-Pugh score at 3 months, the percentage change of bilirubin at 3 months, the absolute spleen volume and the percentage change of craniocaudal spleen size and spleen volume (%-SV) at 3 months post-TARE. No significant associations were observed for other baseline risk factors, including the number of tumor lesions, tumor morphology, tumor growth characteristics, baseline BCLC stage, or embolization strategy (lobar, segmental, or subsegmental TARE), with progression-free survival. Notably, the hepatic volume at baseline or changes thereof at any time during follow-up were also not associated with overall patient survival. Likewise, TNr, derived from ^99m^Tc-MAA SPECT/CT, showed no significant association with overall survival. The full results of the univariate log-rank test can be found in Supplement Table A.

The features identified in the univariate survival analysis were evaluated for multicollinearity using variance inflation factors (VIFs) and as the MELD-Score at 3 months demonstrated a moderate collinearity (VIF = 5.5) it was excluded from further analysis. The remaining features were then jointly included in a multivariate Cox proportional hazards model to assess their combined prognostic performance. In this multivariate analysis of all identified features listed in Table 2, only the relative change of spleen volume after 3 months remained significantly associated with PFS (HR 1.02 (1.01–1.02, *p* < 0.001). A full summary of the multivariate survival analysis can be found in Table 2.

**Table 2:** Results from the uni- and multivariate Cox proportional hazard analysis including features significantly associated with progression free survival of patients (*p* < 0.1 for univariate preselection). While baseline established risk scores such as MELD and Child-Pugh score at 3 months or the laboratory values such as ALT at baseline or the relative decline in bilirubin at three months were significant in the univariate analysis, only for the relative decline of spleen volume %-SV) after 3 months the hazard ratio remains significantly different from one in the multivariate analysis. Legend: HR = hazard Ratio, CI = Confidence Interval, p = p-value, SV = spleen Volume, ALT = alanine aminotransferase, CC-S = cranio-caudal spleen size

Feature	Univariate Cox Proportional Hazard			Multivariate Cox Proportional Hazard		
	HR	CI	*p*	HR	CI	*p*
MELD Score Baseline	1.07	0.99–1.17	**0.099**	1.02	0.96–1.08	0.481
ALT [U/l] Baseline	0.99	0.99–1.00	**0.068**	1.00	0.99–1.00	0.163
Child-Pugh Score 3 months	1.67	1.10–2.52	**< 0.001**	1.18	0.86–1.63	0.308
%-Change Bilirubin 3 months	1.01	1.00–1.01	**0.047**	1.00	0.99–1.01	0.470
Spleen Volume 3 months [ml]	1.01	1.01–1.02	**< 0.001**	1.00	0.99–1.00	0.100
%-Change CC-S at 3 months	1.10	1.00–1.21	**0.004**	1.03	0.97–1.10	0.350
%-SV 3 Months	1.04	1.03–1.05	**< 0.001**	1.02	1.01–1.02	**< 0.001**

### Changes in spleen volume predictive for 1-year PFS

**Fig. 2:**
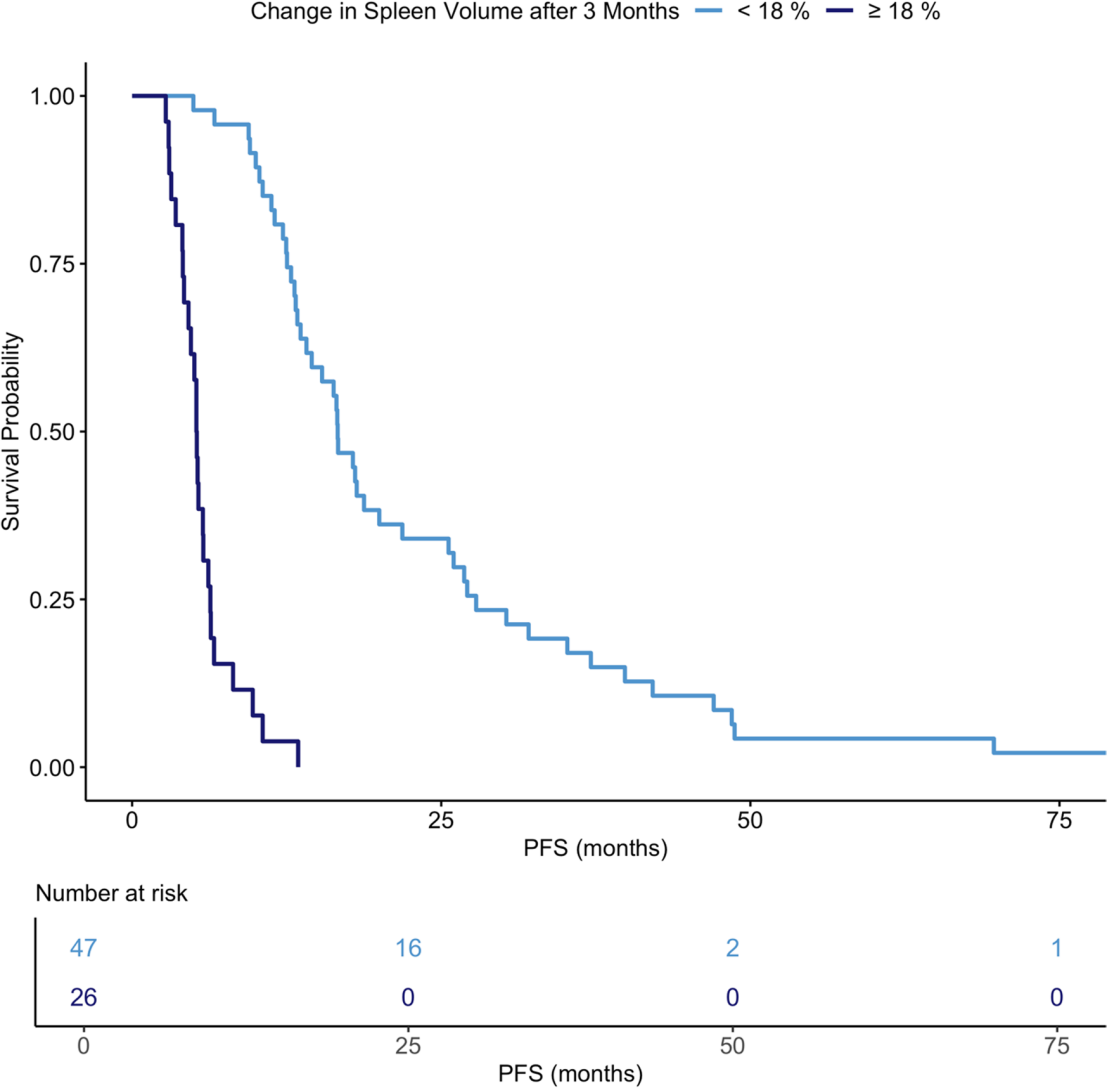
Kaplan Meier survival plot shows progression free survival of patients with an increase in spleen volume 3 months post treatment of ≥ 18% (dark blue) and those with < 18% (light blue)

Based on these results, the prognostic performance of changes in spleen volume after 3 months for patient survival was further investigated. Patients were stratified into two groups based on PFS of more or less than 12 months, as the 1-year PFS milestone represents a key benchmark in HCC trials for evaluating treatment efficacy and predicting long-term outcomes [32]. This cutoff also corresponds to the median time to progression in our study (12.6 months (IQR 5.8–20.0 months)). ROC analysis provided a specific threshold of 18%-increase in spleen volume after three months.

to distinguish between patients experiencing an oncologic progress within a year after treatment start and those who did not (ROC area under the curve 0.86, 95%-CI: 0.76–0.95). Figure 2 shows the Kaplan Meier plot illustrating the differences of PFS for patients with respect to a high or low increase of spleen volume after 3 months.

**Fig. 3:**
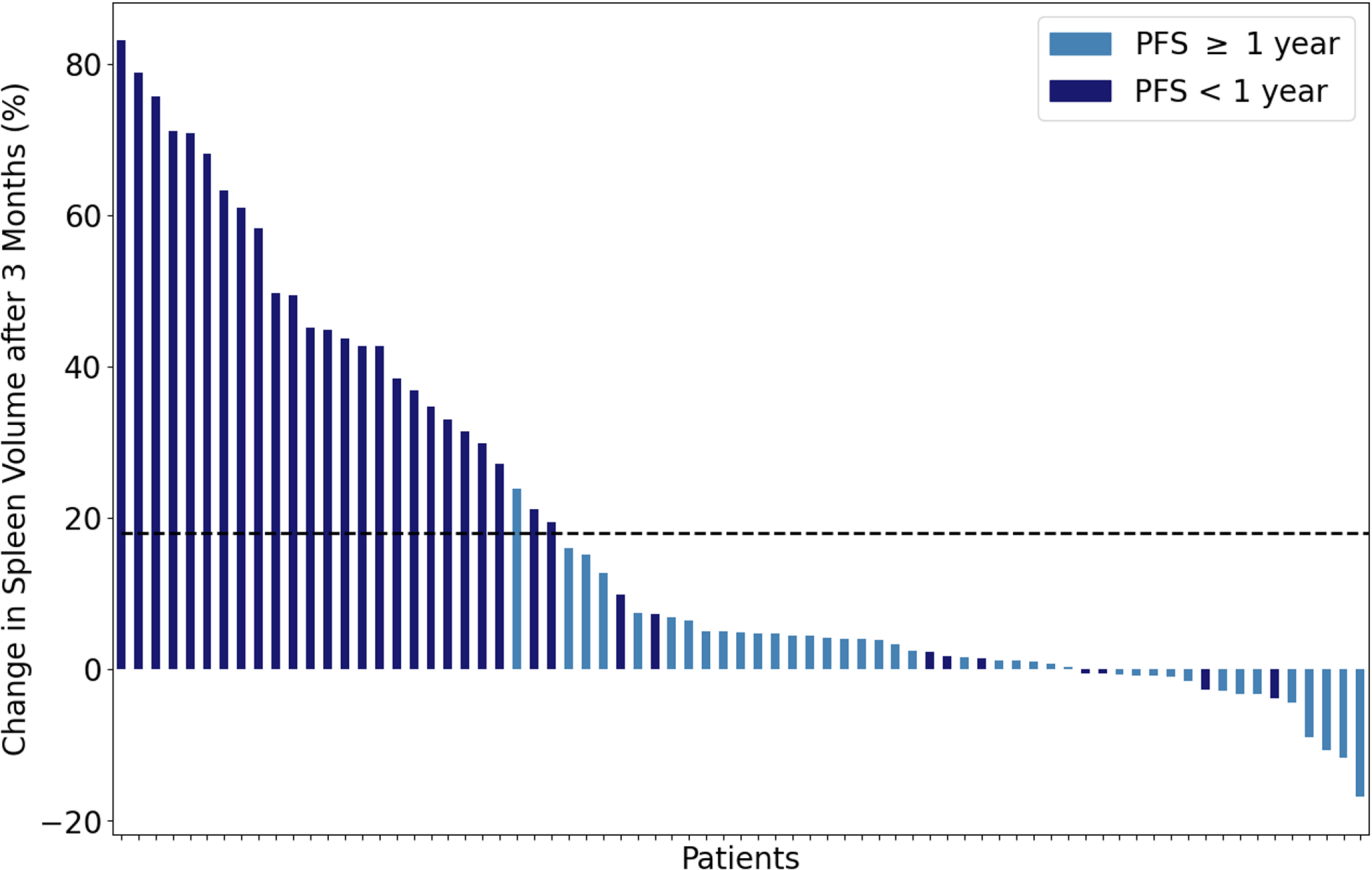
Waterfall plot illustrating the relative change in spleen volume (%-SV) at 3 months for all patients. The dashed line indicates the determined 18% decline threshold. With this, a high discrimination between the patients experiencing oncologic progression within a year and those who did not can be achieved (ROC-AUC: 0.86 (95%-CI; 0.76-0.0.76.0.95), sensitivity 0.74, specificity 0.97)

As shown in Fig. 3, using a cut-off of 18%-increase in spleen volume a high discrimination between the two patient groups can be achieved with a sensitivity of 0.74 and a specificity of 0.97.

## Discussion

In HCC, especially under TARE, treatment planning must balance efficacy, toxicity, and survival prediction, given the frequent presence of cirrhosis and limited hepatic reserve [4, 33, 34]. As a locoregional therapy, TARE targets tumors via the hepatic artery while largely sparing healthy liver tissue [8, 35]. Imaging-derived biomarkers - particularly changes in splenic volume - have emerged as promising, non-invasive tools for early risk stratification and personalized treatment planning [2, 36]. In this study, we analyzed imaging data from 73 consecutive patients with HCC who underwent TARE to evaluate the prognostic value of splenic and hepatic volumetric changes. In addition to imaging parameters, routinely used clinical and laboratory markers—including AST, ALT, GGT, bilirubin, albumin, and alpha-fetoprotein (AFP) as well as established liver function scores such as the ALBI grade, Child-Pugh score, and MELD score were assessed for their association with progression-free survival. Univariate Cox proportional hazards analysis identified several post-treatment parameters significantly associated with progression-free survival (PFS), including changes in splenic volume at 3 months and dynamic hepatic function markers, such as the percentage change in bilirubin. Among baseline variables, only serum ALT levels and the MELD score were predictive of PFS. In contrast, baseline liver volume, tumor burden, and clinical staging systems—including the Child-Pugh score and ALBI grade—were not significantly associated with PFS.

Due to the absence of a widely accepted prognostic model for this patient population, and the generally limited prognostic performance of established clinical parameters post TARE, we did not assess the added value of changes in liver and spleen volume relative to a reference model. Instead, we first performed univariate testing of all features and subsequently included only significant parameters in a multivariate Cox proportional hazards model. In this multivariate analysis, a 3-month increase in splenic volume emerged as the only independent predictor of progression-free survival (PFS), suggesting that early volumetric changes in the spleen may reflect biologic processes associated with disease progression. Stratification by a 12-month PFS threshold—a clinically relevant milestone in HCC trials - identified an 18% increase in splenic volume as the optimal cut off to differentiate between patients with early progression and those with sustained disease control. This threshold demonstrated strong discriminatory performance, with a sensitivity of 0.74 and a specificity of 0.97, supporting its potential as a non-invasive imaging biomarker for early risk stratification following TARE.

These findings are consistent with previous studies showing that splenic volume and its dynamic changes serve as robust prognostic indicators in HCC patients undergoing transarterial chemoembolization (TACE), both in the first-line and adjuvant settings [2, 37]. Furthermore, splenic volume changes have also been observed in the context of systemic therapies. In patients receiving immune checkpoint inhibitors, higher baseline splenic volume and progressive increases have been associated with shorter PFS and overall survival [38]. Similarly, Chen et al. reported that splenic volume increases in HCC patients treated with sorafenib correlated with earlier disease progression [39].

In contrast, although some traditional liver function parameters - such as baseline ALT and MELD scores, changes in MELD and Child-Pugh scores at 3 months, and bilirubin dynamics - showed associations in univariate analysis, none remained significant in the multivariate model. This highlights the limitations of conventional scoring systems like the Child-Pugh score, which in addition includes subjective elements such as ascites and encephalopathy that are prone to interobserver variability and may be influenced by non-hepatic factors [40, 41]. The MELD score, although widely adopted, relies solely on bilirubin, INR, and creatinine, failing to fully capture the complexity of hepatic function - particularly in patients with renal impairment unrelated to liver disease [42]. Although the BCLC staging system is widely used to guide treatment allocation in HCC, it has recognized limitations. Notably, BCLC stage B encompasses a highly heterogeneous patient population with considerable variability in tumor burden, liver function, and clinical course, which is not adequately captured by the current classification [43]. Moreover, bilirubin and albumin levels are susceptible to extrahepatic influences including hemolysis, inflammation, nutritional status, and circulatory alterations, limiting their specificity and sensitivity [44]. The craniocaudal spleen diameter as simple one-dimensional marker showed associations in univariate analysis, but did not retain prognostic significance in the multivariate model. Similarly, TNr, derived from ^99m^Tc-MAA SPECT/CT, was not significantly associated with overall survival. Although ^99m^Tc-MAA is routinely used for personalized dosimetry and treatment planning and widely applied as a surrogate for subsequent ^90^Y microsphere distribution [45, 46], its predictive performance is limited. Reported discrepancies between pre-therapeutic ^99m^Tc-MAA uptake and post-therapeutic ^90^Y microsphere distribution have been attributed to differences in flow dynamics, catheter positioning between simulation and treatment, and MAA particle aggregation and size heterogeneity, all of which may reduce the accuracy of dose surrogacy [47–49].

By comparison, three-dimensional volumetric assessment demonstrated superior precision and reproducibility, allowing for the reliable detection of even subtle post-treatment increases. This is particularly relevant in the context of TARE, where splenic enlargement may reflect subclinical hepatitis, subsequent parenchymal contraction, and the development of portal hypertension, ultimately resulting in secondary splenic congestion [18, 50]. In patients with unresectable HCC, the optimal timing of transition from locoregional to systemic therapy remains challenging. Prolonged continuation of TARE may delay or even preclude treatment escalation due to declining liver function [51]. In this setting, splenic volume may serve as an underutilized imaging biomarker to identify patients at increased risk of hepatic decompensation and to guide closer follow-up and interdisciplinary decision-making.

Several limitations of this study should be acknowledged. First, the single-center, retrospective design may introduce selection and information biases. Second, the absence of a control group limits the ability to account for potential confounding variables influencing splenic volume changes. Third, the lack of established reference values for splenic volume dynamics hampers precise interpretation of volumetric thresholds associated with prognosis, particularly in relation to shortened progression-free survival. Fourth, patient-specific post-therapeutic dosimetry data, including tumor and non-tumorous liver absorbed dose, were not consistently available and therefore could not be incorporated into the present analysis. To partially address this limitation, pre-therapeutic Tc-99 m-MAA SPECT/CT was used as a surrogate to approximate microsphere distribution by deriving tumor-to-normal liver uptake ratios; however, such metrics cannot replace quantitative post-treatment absorbed dose measurements. Accordingly, studies integrating post-therapy ^90^Y imaging with voxel-based dosimetry are needed to better delineate the relationship between absorbed dose and spleen volume changes. Moreover, prospective multicenter studies incorporating standardized patient-specific dosimetry, is warranted to evaluate splenic volume dynamics as an independent biomarker, establish robust cutoff values, and validate its utility for risk stratification and treatment optimization in this patient population.

## Conclusion

Our findings highlight the feasibility and clinical relevance of integrating automated splenic volumetry into routine imaging workflows. This imaging biomarker may support clinical decision-making and follow-up in patients undergoing TARE by enabling early identification of individuals at increased risk. Notably, significant post-treatment volume increases could prompt closer monitoring and timely adaptation of subsequent therapy to mitigate hepatic decompensation. Our retrospective analysis demonstrates that an ≥ 18% increase in spleen volume at 3 months post-TARE is a readily accessible imaging-derived predictor of reduced progression-free survival. CT-based automated volumetric measurements outperformed the traditional manual long axis diameter and conventional liver function parameters in prognostic accuracy. However, in the absence of patient-specific post-therapeutic dosimetry, prospective multicenter studies incorporating standardized absorbed dose assessment are required to validate splenic volumetry, establish robust cutoff values, and further clarify its role in risk stratification and treatment optimization for HCC patients undergoing TARE.

## Supplementary Information

Below is the link to the electronic supplementary material.

Supplementary Material 1

## Data Availability

Data generated or analyzed during the study are available from the corresponding author by request.
