# Supplementary material for: Fully automated spleen segmentation predicts progression-free survival in HCC patients following transarterial radioembolization
Source: Eur J Nucl Med Mol Imaging. 2026 Feb 17;53(6):3904–14. doi: 10.1007/s00259-026-07792-8 (PMC13121415; doi:10.1007/s00259-026-07792-8)
Supplement: Supplementary file 1 — Supplementary Material 1 [file 259_2026_7792_MOESM1_ESM.docx]

**SUPPLEMENTAL MATERIAL**

| Feature | HR | 95%-CI | P | Feature | HR | 95%-CI | p |
| --- | --- | --- | --- | --- | --- | --- | --- |
| LV Baseline [ml] | 1.00 | 0.99-1.01 | 0.516 | ALBI Score Baseline | 0.91 | 0.55-1.51 | 0.720 |
| LV 3 Months [ml] | 1.00 | 0.99-1.00 | 0.205 | ALBI Score Baseline | 0.91 | 0.55-1.51 | 0.720 |
| %-LV 3 months | 1.01 | 0.99-1.03 | 0.257 | Albumin Baseline [g/dl] | 0.90 | 0.65-1.22 | 0.504 |
| SV Baseline [ml] | 1.00 | 0.99-1.00 | 0.180 | Albumin 3 Months [g/dl] | 0.70 | 0.42-1.16 | 0.164 |
| SV 3 Months [ml] | 1.00 | 1.01–1.02 | **<0.001** | %-Albumin 3 Months | 0.99 | 0.98-1.02 | 0.830 |
| %-SV 3 Months | 1.04 | 1.03-1.05 | **<0.001** | Bilirubin Baseline [mg/dl] | 1.19 | 0.70-2.04 | 0.515 |
| CC-S [cm] | 1.05 | 0.95-1.15 | 0.341 | Bilirubin 3 Months [mg/dl] | 1.23 | 0.95-1.61 | 0.108 |
| CC-S 3 Months [cm] | 1.06 | 0.96-1.17 | 0.241 | %-Bilirubin 3 Months | 1.00 | 1.00-1.01 | 0.047 |
| %-CC-S 3 Months | 1.10 | 1.00-1.21 | **0.040** | AST [U/l] | 0.99 | 0.96-1.01 | 0.650 |
| TV Baseline [ml] | 0.99 | 0.99-1.00 | 0.398 | AST 3 Months [U/l] | 0.99 | 0.99-1.00 | 0.399 |
| Tumor Lesions ≥2 | 1.05 | 0.66-1.68 | 0.843 | %-AST 3 Months | 0.99 | 0.99-1.01 | 0.893 |
| Number of Tumor Lesions | 1.05 | 0.97-1.14 | 0.187 | ALT Baseline [U/l] | 0.99 | 0.99-1.00 | **0.068** |
| Tumor Morphology | 1.02 | 0.81-1.29 | 0.840 | ALT 3 Months [U/l] | 0.99 | 0.99-1.00 | 0.109 |
| Embolization strategies | 1.05 | 0.79-1.56 | 0.827 | %-ALT 3 Months | 1.00 | 0.99-1.01 | 0.954 |
| TNr | 1.02 | 0.89-1.16 | 0.790 | GGT Baseline [U/l] | 0.99 | 0.99-1.00 | 0.669 |
| ^90^Y TARE Activity [GBq] | 0.87 | 0.69-1.09 | 0.214 | GGT 3 Months [U/l] | 0.99 | 0.99-1.00 | 0.664 |
| LeLu-Shunt [%] | 0.4 | 0.15-15.3 | 0.436 | %-GGT 3 Months | 0.99 | 0.99-1.01 | 0.466 |
| Line of Therapy | 1.12 | 0.70-1.80 | 0.628 | AFP Baseline [ng/ml] | 0.99 | 0.99-1.01 | 0.821 |
| Liver Cirrhosis | 1.44 | 0.83-2.49 | 0.191 | AFP 3 Months [ng/ml] | 1.00 | 0.99-1.01 | 0.470 |
| BCLC Stages | 1.05 | 0.54-2.04 | 0.887 | %-AFP 3 Months | 0.99 | 0.99-1.00 | 0.198 |
| Age [yeaers] | 1.01 | 0.98-1.03 | 0.656 | WBC Baseline [1000/l] | 0.96 | 0.851.07 | 0.445 |
| Height [m] | 1.01 | 0.99-1.02 | 0.178 | WBC 3 Months [1000/l] | 0.98 | 0.88-1.10 | 0.792 |
| Weight [kg] | 0.99 | 0.98-1.01 | 0.657 | %-WBC 3 Months | 1.00 | 0.99-1.01 | 0.209 |
| BMI | 0.97 | 0.92-1.02 | 0.228 | HB Baseline [g/dl] | 0.97 | 0.87-1.10 | 0.714 |
| Sex | 1.35 | 0.77-2.37 | 0.295 | HB 3 Months [g/dl] | 0.98 | 0.85-1.12 | 0.768 |
| Hypertension | 0.84 | 0.52-1.35 | 0.466 | %-HB 3 Months | 1.00 | 0.98-1.02 | 0.910 |
| Diabetes Mellitus | 0.81 | 0.50-1.31 | 0.393 | PLT Baseline [1000/l] | 0.99 | 0.99-1.00 | 0.339 |
| MELD Score Baseline | 1.07 | 0.99-1.17 | **0.099** | PLT 3 Months [1000/l] | 0.99 | 0.99-1.00 | 0.421 |
| MELD Score 3 Months | 1.09 | 1.02–1.15 | **0.007** | %-PLT 3 Months | 1.00 | 0.99-1.01 | 0.693 |
| Child-Pugh Score Baseline | 1.52 | 0.65-3.56 | 0.336 | Age ≥ 65 years | 1.35 | 0.74-2.45 | 0.328 |

**Supplement Table A:** Results from univariate Cox Proportional Hazard Analysis for all features tested in this study. For univariate pre-selection of parameters, a level of significance of p<0.1 was chosen. Percentage change of features is denotes as „%-“. Abbreviations: HR = Hazard Ratio, CI = Confidence Interval, p = p-value, LV = Liver Volume, SV = Spleen Volume, CC-S = cranio-caudal Spleen Size, TV = Tumor Volume, TNr = tumor-to-normal liver ratio, ALT = Alanine Tansaminase, AST = Aspartate Aminotransferase, GGT = Gamma-Glutamyl Transferase, AFP = Alpha-fetoprotein, WBC = White Blood Cell Count, PLT = Platelet Count, HB = Hemoglobin.


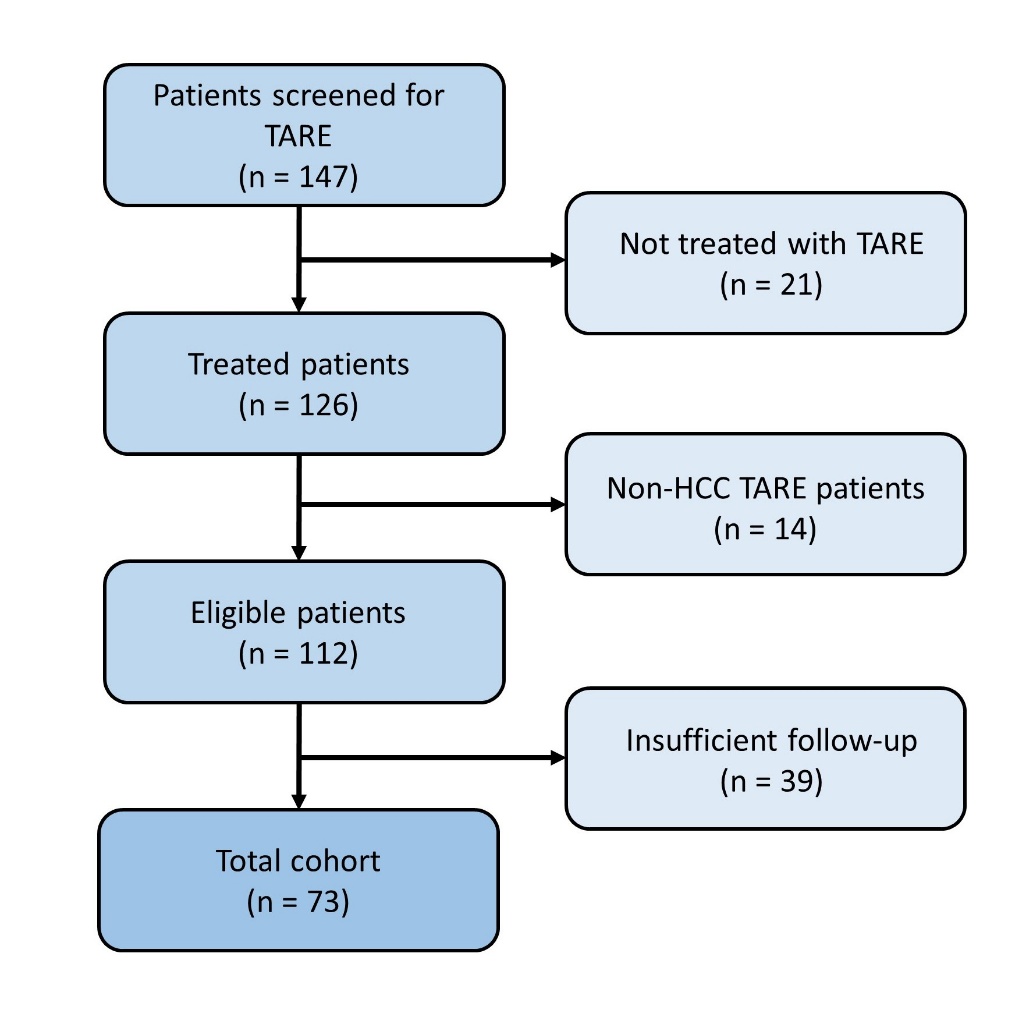


**Supplementary figure S1.** Inclusion flowchart. TARE: Transarterial radioembolization; Non-HCC: Patients who underwent TARE for clinical indications other than hepatocellular carcinoma.
